# CAREGIVING IMPACT ON MEDICARE OLDER ADULTS: RESULTS OF THE US HEALTH AND WELLNESS SURVEY

**DOI:** 10.1093/geroni/igae098.3822

**Published:** 2024-12-31

**Authors:** Lycia Tramujas Vasconcellos Neumann, Kathy Annunziata, Lauren Stratton

**Affiliations:** Alzheimer’s Association, Chicago, Illinois, United States; Oracle Life Sciences, Austin, Texas, United States; Alzheimer’s Association, Chicago, Illinois, United States

## Abstract

As the number of older adults caring for someone with a serious illness increases, there is a growing need to understand better the impact of caregiving on older adults’ health and finances. Based on responses from older Medicare recipients (65+) to the US 2022 National Health and Wellness Survey, an internet-based, cross-sectional survey, this study investigated differences in self-reported health status, healthcare utilization, and out-of-pocket costs between (1) older adults who are caregivers (n = 1,159) and those who are not (n = 11,443), and (2) older caregivers of individuals living with Alzheimer’s disease or other dementias - ADRD (n = 255) and those caring for an adult with other conditions (n = 904). Results were weighted to the population by sex, age, and race/ethnicity. Caregiver and non-caregiver cohorts had comparable median and mean ages. Compared with older non-caregivers, older caregivers had significantly higher comorbidities and significantly lower global health, physical health, and mental health T scores (p< 0.05). There were no significant differences in reported use of ER, hospitalizations, and median number of medical visits in the six months before the survey. However, older caregivers reported higher mean out-of-pocket Rx expenses than non-caregivers ($44.51 vs. $34.02). Comparative analyses of older dementia caregivers with non-dementia caregivers did not indicate significant differences in health and financial impact. In conclusion, caregiving impacts older adults’ health in various ways, and health plans and providers should acknowledge and consider the impact of caregiving when assessing older adults’ health conditions, managing and coordinating their care.

